# The Posterior Extension of the Palatal Rugae as an Anatomical Constraint for Soft Tissue Grafts in a Saudi Arabian Population

**DOI:** 10.7759/cureus.32731

**Published:** 2022-12-20

**Authors:** Abdulsalam Alshammari, Fathima Fazrina Farook, Lulu A Alyahya, Maha N AlHarbi, Norah N Alazaz, Lubna AlKadi, Farraj Albalawi, Ali A Aboalela

**Affiliations:** 1 College of Dentistry, King Saud Bin Abdulaziz University for Health Sciences, Riyadh, SAU; 2 Research and Development, King Abdullah International Medical Research Center, Riyadh, SAU; 3 Dental Hospital, Ministry of National Guard for Health Affairs, Riyadh, SAU

**Keywords:** soft tissue grafts, saudi arabian population, rugae patterns, posterior extension, palatal rugae

## Abstract

Background

The purpose of this study was to investigate the distal extension of the palatal rugae area as an anatomical constraint on the harvesting of palatal soft tissue grafts in a Saudi Arabian population. Additionally, factors that could affect or predict the extension were considered.

Methods

Three hundred seventy-four (374) dental casts from Saudi nationals currently residing in Riyadh (170 males and 204 females) were included. Two independent observers used a standardized probe to measure the posterior extent of the rugae on each stone cast bilaterally on a horizontal base. A sharp graphite pencil was used to mark the measurements from the origin of the rugae to their terminal ends on the cast, and a magnification lens was used to identify them. Using this technique, the most posterior extension of the rugae was marked and then analyzed. The normal approximation test for binomial distribution was used to determine the proportion of the subjects with rugael extensions beyond the mesial end of the upper second premolar, and logistic regression was used to see the association of this extension with other factors.

Results

The asymptotic chi-squared (p = 0.0002) McNemar tests revealed that the posterior distal extension of the rugae was not the same on both sides. A normal approximation test for the left side with 95% confidence intervals (CIs) with the “rugael extension proximal to the mesial end of the upper second premolar” category considered “success” found that the proportion of upper second premolars with rugael extensions proximal to the mesial end was not significantly different to the proportion of rugael extensions beyond the mesial end of the upper second premolars (95% CI: 48.69%-58.79%, p = 0.147).

Conversely, the proportion of the upper second premolars with rugael extensions proximal to the mesial end was significantly lower than that beyond the mesial end on the right (95% CI: 35.92%-45.89%, p = 0.00004). Gender, age, and palatal shape did not significantly affect the posterior extension of palatal rugae.

Conclusions

The palatal rugae on the left side of a sample of the Saudi Arabian population do not considerably extend beyond the upper second premolar mesial aspect, which may provide reliable soft tissue grafts for esthetic mucogingival surgery.

## Introduction

Palatal rugae are elevations found on the anterior part of the palatal mucosa, behind the incisive papilla. Rugae palatinae and plicae palatinae transversae are synonyms of palatal rugae. Rugae are composed of three to seven ridges radiating from the incisive papilla (just anterior to the incisive foramen) tangentially [1-3].

Numerous classification methods exist for palatal rugae based on number, extent, shape, and type [4]. In addition to serving as a reference landmark in dentistry, they also support the identification of submucosal clefts. Anatomically, they are positioned to aid in oral swallowing, suction in children, food crushing, taste perception, and speech, especially in the production of “s” and “sh” phonemes. Rugae influence the mechanical properties of food and the position of the tongue [4]. Palatal rugae are highly individual and remain consistent in shape throughout life, and palatal rugae can be used as an alternative method of postmortem identification [2-5]. Studies of palatal rugae have been conducted for a range of purposes, including anthropology, genetics, comparative anatomy, orthodontics, forensic odontology, and prosthodontics [1,6-9].

Soft tissue graft donor sites are limited by the posterior extent of the rugae. Oral and periodontal soft tissue augmentation procedures typically utilize free epithelialized grafts and subepithelial connective tissue grafts from the hard palate and tuberosity [10-14]. Several procedures can be performed, including the augmentation of keratinized tissues, the treatment of gingival recessions, and the correction of localized defects on the alveolar ridge [15,16]. The most preferred source for such grafts is the keratinized mucosa of the maxillary premolars attached to the palatal mucosa [10].

Since rugae tend to retain their particular shape and will reestablish if incised from a free gingival graft, they result in a long-lasting esthetically unpleasant appearance if removed within a soft tissue graft. Palatal rugae present an anatomical barrier to free gingival grafts due to their unesthetic appearance. Considering the posterior extension of the palatal rugae, the hard palate may not be a suitable soft tissue donor site for mucogingival procedures [17,18]. It is crucial to understand the extent of palatal rugae posteriorly because that can limit soft tissue palatal grafts at the anterior level.

Despite the significance of such knowledge, there is insufficient literature on the distal extent of rugae. To date, only one study investigated the distal extension of the rugae area in a Jordanian (Middle Eastern) population [19]. Interestingly, rugae patterns differ among ethnicities, and studies demonstrated a substantial correlation between various characteristics, including the length, form, orientation, and unification of rugae and ethnicity [1], as well as a substantial correlation between rugae shapes and ethnicity [20-27].

It is beneficial to determine the distal extent of rugae in a Saudi Arabian population due to the variation in patterns between ethnic groups. Research on this population has so far focused on rugae characteristics, individuality, and gender specificity [24,28,29]. The purpose of this study was to determine the posterior extent of the rugae area in a Saudi Arabian population as a surgical limitation affecting the decision to harvest soft tissue grafts from the palatal area.

## Materials and methods

This cross-sectional study was conducted at the College of Dentistry, King Saud Bin Abdulaziz University for Health Sciences, between August 2021 and August 2022. Strengthening the Reporting of Observational Studies in Epidemiology (STROBE) was followed as a cross-sectional checklist. The college obtained informed consent from all participants and parents/legal guardians of participants to use casts and investigations for research purposes and study participation prior to any dental treatment.

Sample size estimation

Using a two-sided Z-test with S(P0) to estimate the standard deviation and an alpha level of 0.050, a sample size of 374 achieved a 93% power to detect a difference (P1-P0) of 0.1. This result is based on the assumption that the population proportion under the null hypothesis is 0.5000. Power Analysis and Sample Size (PASS) 2020 version 20.0.4 (Number Cruncher Statistical Systems (NCSS), LLC, Kaysville, UT, USA) was used to calculate the sample size.

In total, 500 dental casts from the database of the college were evaluated. The dental casts were selected from the participant database. The eligibility criteria were as follows: Saudi nationals, age above 16 years, currently living in Riyadh, and with full maxillary dentition (except for third molars).

Casts were excluded if patients had a history of a disease, surgery to the palate or tuberosity, any dental appliances in the maxilla, previous orthodontic treatment, extracted or congenitally missing premolars, medications that may affect the periodontal soft tissues, or malpositions or misalignments of the maxillary posterior teeth.

There were a total of 374 eligible casts (170 (45.45%) males and 204 (54.55%) females) from the main population of Saudi Arabia. Every cast was given a serial number, and the gender and age of the patient were also marked on each cast. Institutional Review Board approval was obtained from King Abdullah International Medical Research Center for the study (IRB number: NRC21R/246/06).

**Figure 1:**
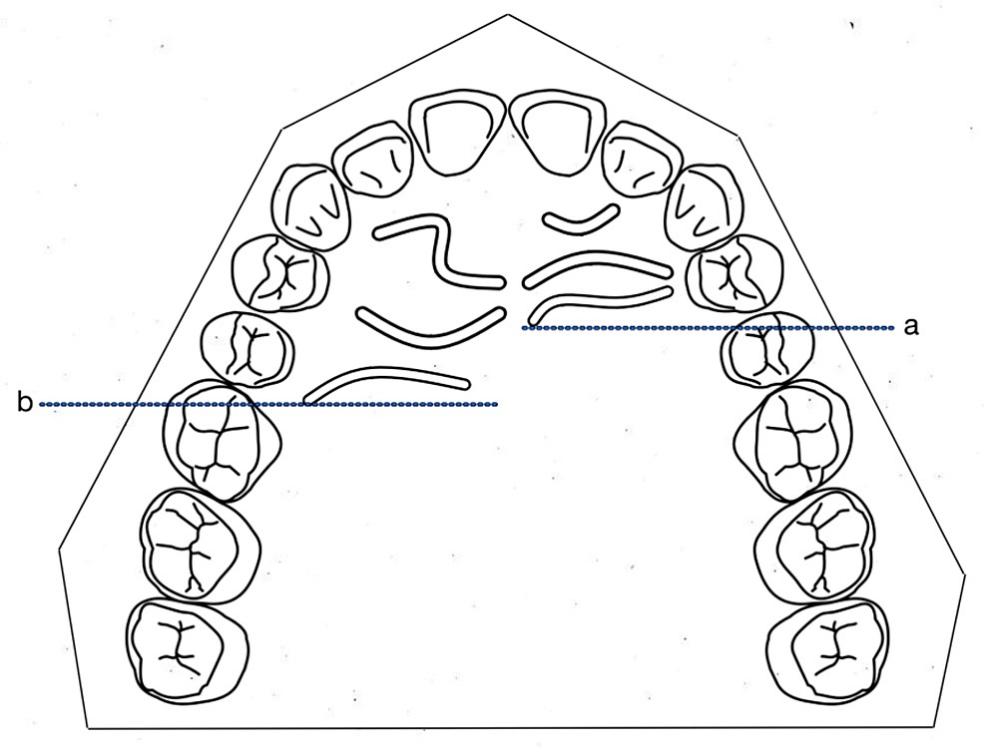
a: Recording parameter of the distal extension of rugae on the left side of the palate. b: Recording parameter of the distal extension of rugae on the right side of the palate.

A standardized probe was used to measure the posterior extent of the rugae. Two independent observers (LA and MA) took all the measurements in a well-illuminated room. Each stone cast was evaluated bilaterally on a horizontal base. A sharp graphite pencil was used to mark the measurements from the origin of the rugae to their terminal ends on the cast (Figure 1), and a magnification lens was used to identify them. Using this technique, the most posterior extension of the rugae was marked and analyzed.

Statistical analysis

The proportion of participants with rugael extensions beyond the mesial end of the upper second premolar was estimated using the normal approximation test with a 95% confidence interval (CI) on a sample of 374 subjects. A “rugael extension proximal to the mesial end of the upper second premolar” was considered successful. Logistic regression tests were used to check for significant associations with potentially contributing factors including age, gender, and jaw type (U-shaped and V-shaped palate). The agreement between the two raters was tested using Cohen’s Kappa inter-rater agreement. Analysis was performed using Number Cruncher Statistical Systems (NCSS) 2020 statistical software, statistical hypothesis tests were two-tailed, and the criteria for statistical significance were p < 0.05.

## Results

The study included 374 participants in total. The rugae extended beyond the mesial end of the upper second premolar on the right side of the palate in 221 (59.09%) participants. Among the rugae on the left side, fewer than half (n = 173, 46,25%) extended beyond the upper second premolar’s mesial end. The implication is that on the left side of the palate, a greater percentage of the rugae did not extend beyond the mesial end of the upper second premolar. The distribution of the posterior furthermost extension of palatal rugae on both the right and left sides of the participants is shown in Table 1.

**Table 1:** Percentage distribution of the posterior extension of the palatal rugae in relation to the teeth and according to the side, gender, and palatal shape. LS: left side, RS: right side

	Palatal shape				Gender				Sides	
Posterior most extension of the rugae	U shape/LS (number (%)) (306 (81.81))	U shape/RS (number (%)) (306 (81.81))	V shape/LS (number (%)) (68 (18.18))	V shape/RS (number (%)) (68 (18.18))	Female/LS (number (%)) (204 (54.55))	Female/RS (number (%)) (204 (54.55))	Male/LS (number (%)) (170 (45.45))	Male/RS (number (%)) (170 (45.45))	Left (number (%))	Right (number (%))
Mesial of the first premolar	13 (4.24)	8 (2.61)	2 (2.94)	1 (1.47)	7 (3.43)	4 (1.96)	8 (4.71)	5 (2.94)	15 (4.01)	9 (2.41)
Distal of the first premolar	35 (11.44)	25 (8.17)	11 (16.18)	11 (16.18)	25 (12.25)	18 (8.82)	21 (12.35)	18 (10.59)	46 (12.30)	36 (9.63)
Mesial of the second premolar	119 (38.89)	88 (28.76)	21 (30.88)	20 (29.41)	80 (39.22)	60 (29.41)	60 (35.29)	48 (28.24)	140 (37.43)	108 (28.88)
Distal of the second premolar	109 (35.62)	148 (48.37)	27 (39.71)	25 (36.76)	75 (36.76)	95 (46.57)	61 (35.88)	78 (45.88)	136 (36.36)	173 (46.26)
Mesial of the first molar	26 (8.49)	34 (11.11)	7 (10.29)	11 (16.18)	14 (6.86)	25 (12.25)	19 (11.76)	20 (11.76)	33 (8.82)	45 (12.03)
Distal of the first molar	4 (1.30)	3 (0.98)	0 (0)	0 (0)	3 (1.47)	2 (0.98)	1 (0.59)	1 (0.59)	4 (1.07)	3 (0.8)
Mesial of the second molar	0 (0)	0 (0)	0 (0)	0 (0)	0 (0)	0 (0)	0 (0)	0 (0)	0 (0)	0 (0)
Total	306 (100)	306 (100)	68 (100)	68 (100)	204 (100)	204 (100)	170 (100)	170 (100)	374 (100)	374 (100)

**Figure 2:**
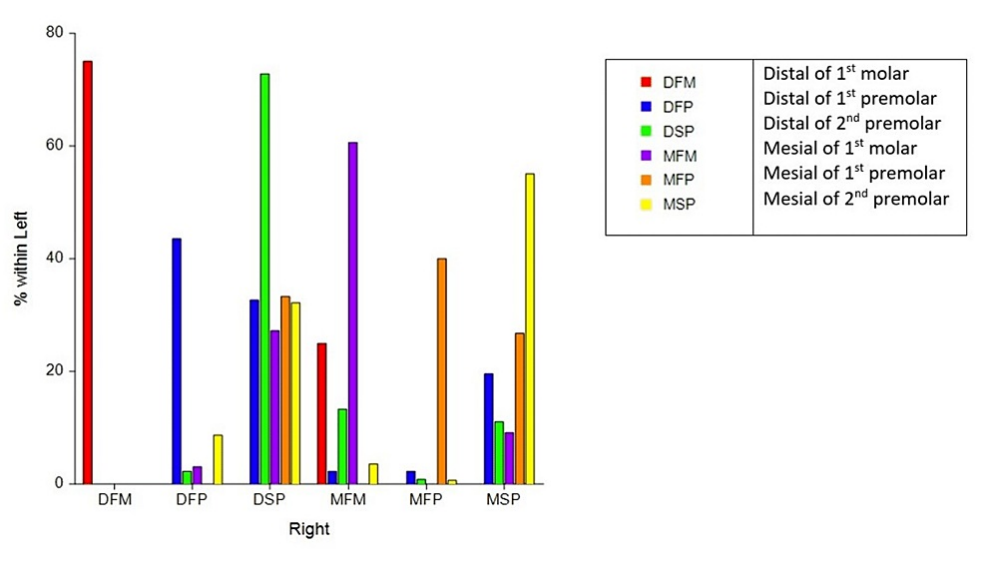
Percentage of bilateral symmetry in the distal rugael extension (left matching with the right as a percentage). DFM: distal of the first molar, DFP: distal of the first premolar, DSP: distal of the second premolar, MFM: mesial of the first molar, MFP: mesial of the first premolar, MSP: mesial of the second premolar

The asymptotic chi-squared (p = 0.0002) McNemar tests to assess the bilateral symmetry in the posterior extension of the palatal rugae indicated that there is enough evidence to reject the null hypothesis. The distal extension of the rugae is not the same on both sides. The trend has been depicted in Figure 2. The X-axis represents the site of the distal-most extension of rugae on the right side of the palate. The Y-axis represents the percentage symmetry with the left side of the palate. As seen in the figure, the bilateral symmetry of the distal-most extension of palatal rugae was greater than 60% for the distal first molar, the mesial of the first molar, and the distal of the second premolar. Even if the distal extension of the palatal rugae on the right side did not completely match the left side of the palate, it would still match a closer location and not vary greatly from the right side.

For the right side

The posterior extension of the palatal rugae in 153 (40.90%) out of the 374 randomly selected participants was proximal to the mesial aspect of the second premolar and 221 (59.09%) beyond the mesial end of the second premolar.

The normal approximation test performed with 95% CI with the “rugael extension proximal to the mesial end of the upper second premolar” considered to be the category of “success” revealed that the proportion with rugael extensions proximal to the mesial end of the upper second premolar was significantly lower than the proportion beyond the mesial end of the upper second premolar (95% CI: 35.92%-45.89%, p = 0.00004).

For the left side

Of the 374 randomly selected participants, in 201 (53.74%), the posterior extension of the palatal rugae was proximal to the mesial aspect of the second premolar, and 173 (46.25%) had the rugae extension beyond the mesial end of the second premolar.

The normal approximation test performed with 95% CI with the “rugael extension proximal to the mesial end of the upper second premolar” considered to be the “success” category revealed that the proportion with rugael extensions proximal to the mesial end of the upper second premolar was not significantly different to the proportion beyond the mesial end of the upper second premolar (95% CI: 48.69%-58.79%, p = 0.147).

Logistic regression was performed to determine the effect of age, gender, and palatal shape on the likelihood that participants have the rugael extensions mesial to the second premolar bilaterally. The logistic regression model was not statistically significant (p > 0.05). The overall model fit was 23.78% (Nagelkerke R2) for the left side and 28.81% for the right side of the palate. None of the predictors contributed significantly to the outcome (p > 0.05). There was a good inter-rater agreement between the findings of the two raters with a Kappa coefficient of 0.95.

## Discussion

Recently, numerous studies investigated palatal rugae patterns, form, orientation, and unification in particular racial groups [1,28,30-32]. Palatal rugae in mammalians are specie-specific [33]. Differences in ethnicity are associated with genetic variation [32], distinct patterns of tooth movement brought on by crowding and wear, and changes in the extent of palatal growth and pattern.

To our knowledge, this study is the first to investigate the bilateral symmetry of the distal-most extension of palatal rugae and its extension relative to the teeth as an anatomical constraint for soft tissue graft harvesting in the Saudi Arabian population. In addition, the study also investigated the relationship between gender, age, palatal shape, and rugael distal extension.

We found a right-left asymmetry concerning the posterior-most extension of the palatal rugae, although a pattern existed on both sides (Figure 2). Interestingly, in contrast to the right side, which showed a significantly lower proportion of rugael extension proximal to the mesial end of the upper second premolar, the left side of the palate revealed no significant difference between the proportion of rugael extension proximal to the mesial of the upper second premolar versus beyond the mesial end of the upper second premolar. No significant relationship between age, palatal shape, and gender, and the palatal rugae extension was found.

The preferred place to harvest grafts is from the canine-premolar region, 8-13 mm from the midpalatal face of each tooth [34]. The greater palatine bundle is less at risk in this location; however, the presence of the rugae increases the chance of an undesirable cosmetic result, especially with free gingival grafts [34]. There is not much information about the effect of transplanting tissue from the rugae-containing anterior palate. Although gingivoplasty was performed two months after surgery, Breault et al. observed palatal folds preserved in free gingival grafts nine years after surgery [35], and Soehren et al. reported two occurrences of retained rugae in 20 investigated free gingival graft biopsies [11]. Despite the efforts to remove them, the transplanted rugae remained a permanent fixture at the recipient site. According to Coslet et al. [10] and Breault et al. [35], the underlying connective tissue determines the features of the epithelium [10,36,37], and because rugae in the palatal donor tissue usually reappear months after treatment, clinical removal does not permanently improve the topography. Grafts must not contain rugae from the anterior region of the palate, according to Wolf and Rateitschak-Pluss [38]. Cohen advised that donor tissue be obtained from the posterior portion of the palate, distant to the anterior rugae, as this region had the least quantity of submucosa and the broadest gingival zone [39]. The fact that rugae extend posteriorly to the premolars, both mesiodistally and mediolaterally, complicates the issue further. These anatomical landmarks should be avoided when harvesting grafts for cosmetic reasons [35].

Although there is sufficient literature illustrating right-left rugae asymmetry concerning shape, number, length, and/or direction [7,24,40], this is the first study to explore the bilateral symmetry of the posterior extension of the palatal rugae. A significant difference between the extension of rugae on either side of the palate was demonstrated in this study, confirming the findings in earlier studies illustrating the asymmetrical nature of the palatal rugae in terms of number, length, shape, and/or direction [41-44]. The assumption is that the Saudi population uses teeth on the right side for mastication more than the left side, resulting in less strength and fewer rugae characteristics. The study by Syed et al. could be an explanation for the reduced distal extension of the palatal rugae on the left side [40].

In contrast to a study in a Jordanian population that found a greater percentage of rugae (90%) extended up to the upper second premolar and 78.3% further extended beyond the mesial aspect of the premolar [19], our findings showed a comparatively lesser percentage; fewer than half (n = 173, 46.25%) extended beyond the upper second premolar’s mesial end on the left side and 59.09% on the right side of the palate. However, the Jordanian study had not taken the sides into account in their evaluation [19]. To avoid the unattractive implantation of palatal rugae in the Saudi Arabian population, obtaining a soft tissue graft from the region on the left side may be the most suitable option.

Contrary to the literature suggesting moderate gender differences in the number, length, size, and direction of rugae, the current study found no significant correlation concerning gender [28,45]. However, the studies did not account for the posterior extension of palatal rugae. Additionally, it was found that there was no significant influence of age or palatal shape on the posterior extension of the rugae. This is similar to the findings of the study by Said et al. [19].

Strengths

An important strength of the study is the exclusion of participants using removable appliances, thereby reducing the possibility of mechanical stress to the palatal mucosa. By doing so, confounding factors and other factors that might influence rugael morphology and the findings of this study were minimized. The sample was primarily composed of people aged 15-48 years, which is the target group for periodontal mucogingival surgery, which mandates collecting soft tissue grafts from the hard palate. The current study also showed an almost similar distribution in gender. The adequate sample size and the use of two raters with good inter-rater reliability are additional strengths, which increase the ability to extrapolate the results to a broader population.

Limitations

In light of the possible regional variations that can occur in Saudi Arabia, multicenter studies may be warranted to increase the evidence further.

Finally, evidence from this study suggests that the left side of the Saudi Arabian hard palate is preferred for harvesting soft tissue grafts over the right side. Rugae only slightly limit the anatomical possibilities for harvesting soft tissue grafts on the left side than the right side of the palate. It is also noteworthy to remember that future research is necessary to study the potential danger of taking soft tissue grafts from deeper regions of the palate and to extrapolate our findings to the Saudi Arabian population. If the Saudi Arabian population’s hard palate is deemed insufficient for collecting soft tissue grafts, other donor sites such as the tuberosity should be considered and researched.

## Conclusions

Within the limitations of this study, the rugae on the left side of the palate in a sample of the Saudi Arabian population do not considerably extend beyond the mesial aspect of the upper second premolar, which may provide reliable soft tissue grafts for esthetic mucogingival surgery. Also, the most posterior extent of the palatal rugae did not significantly correlate with gender, age, or palatal shape.

## References

[1] Kapali S, Townsend G, Richards L, Parish T (1997). Palatal rugae patterns in Australian aborigines and Caucasians. Aust Dent J.

[2] Saadeh M, Ghafari JG, Haddad RV, Ayoub F (2017). Association among geometric configurations of palatal rugae. J Forensic Odontostomatol.

[3] Gibelli D, De Angelis D, Pucciarelli V (2018). Application of 3D models of palatal rugae to personal identification: hints at identification from 3D-3D superimposition techniques. Int J Legal Med.

[4] Sabarigirinathan C, Vinayagavel K, Meenakshi A, Rajendran A (2015). Palatal rugae in forensic odontology-a review. IOSR J Dent Med Sci.

[5] Hemanth M, Vidya M, Prasad N, Karkera BV (2009). Human identification using palatal rugae: manual method. Indian J Forensic Med Toxicol.

[6] Shailaja AM, Romana IR, Narayanappa G, Smitha T, Gowda NC, Vedavathi HK (2018). Assessment of palatal rugae pattern and its significance in orthodontics and forensic odontology. J Oral Maxillofac Pathol.

[7] Saadeh M, Ghafari JG, Haddad RV, Ayoub F (2017). Sex prediction from morphometric palatal rugae measures. J Forensic Odontostomatol.

[8] Fatima F, Fida M (2019). The association between morphological characteristics of palatal rugae and sagittal skeletal patterns. J Pak Med Assoc.

[9] Ali B, Shaikh A, Fida M (2016). Stability of palatal rugae as a forensic marker in orthodontically treated cases. J Forensic Sci.

[10] Coslet JG, Rosenberg ES, Tisot R (1980). The free autogenous gingival graft. Dent Clin North Am.

[11] Soehren SE, Allen AL, Cutright DE, Seibert JS (1973). Clinical and histologic studies of donor tissues utilized for free grafts of masticatory mucosa. J Periodontol.

[12] Sullivan HC, Atkins JH (1968). Free autogenous gingival grafts. I. Principles of successful grafting. Periodontics.

[13] Bernimoulin JP, Lüscher B, Mühlemann HR (1975). Coronally repositioned periodontal flap. Clinical evaluation after one year. J Clin Periodontol.

[14] Harris RJ, Harris AW (1994). The coronally positioned pedicle graft with inlaid margins: a predictable method of obtaining root coverage of shallow defects. Int J Periodontics Restorative Dent.

[15] Studer S, Naef R, Schärer P (1997). Adjustment of localized alveolar ridge defects by soft tissue transplantation to improve mucogingival esthetics: a proposal for clinical classification and an evaluation of procedures. Quintessence Int.

[16] Zucchelli G, Mazzotti C, Bentivogli V, Mounssif I, Marzadori M, Monaco C (2012). The connective tissue platform technique for soft tissue augmentation. Int J Periodontics Restorative Dent.

[17] Remya V, Kishore Kumar K, Sudharsan S, Arun KV (2008). Free gingival graft in the treatment of class III gingival recession. Indian J Dent Res.

[18] Camargo PM, Melnick PR, Kenney EB (2001). The use of free gingival grafts for aesthetic purposes. Periodontol 2000.

[19] Said KN, Abu Khalid AS, Farook FF (2021). Distal extension of palatal rugae as a limitation for donor soft tissue grafts in a Jordanian population: a cross-sectional study. BMC Oral Health.

[20] Patil MS, Patil SB, Acharya AB (2008). Palatine rugae and their significance in clinical dentistry: a review of the literature. J Am Dent Assoc.

[21] Savita JK, Yathindra Kumar BN, Satish G, Divya KT, Ranjitha J, Pujari RK (2016). Prevalence of palatal rugae shapes in Karnataka and Kerala population: a cross-sectional study. J Int Soc Prev Community Dent.

[22] Nayak P, Acharya AB, Padmini AT, Kaveri H (2007). Differences in the palatal rugae shape in two populations of India. Arch Oral Biol.

[23] Rath R, Reginald BA (2014). Palatal rugae: an effective marker in population differentiation. J Forensic Dent Sci.

[24] Mustafa AG, Allouh M, Tarawneh I, Alrbata R (2014). Morphometric analysis of palatal rugae among Jordanians: further evidence of worldwide palatal rugae individuality. Aust J Forensic Sci.

[25] Ibeachu PC, Didia BC, Arigbede AO (2014). A comparative study of palatal rugae patterns among Igbo and Ikwerre ethnic groups of Nigeria: a University of Port Harcourt study. Anat Res Int.

[26] Abdellatif AM, Awad SM, Hammad SM (2011). Comparative study of palatal rugae shape in two samples of Egyptian and Saudi children. Pediatr Dent J.

[27] Adisa AO, Kolude B, Ogunrinde TJ (2014). Palatal rugae as a tool for human identification. Niger J Clin Pract.

[28] El-Banna A, Al-Rousan M, Sheasha GA (2019). A study of palatal rugae patterns and maxillary inter-canine distance in a Jordanian population sample. Arab J Foren Sci Foren Med.

[29] Mustafa AG, Tashtoush AA, Alshboul OA, Allouh MZ, Altarifi AA (2019). Morphometric study of the hard palate and its relevance to dental and forensic sciences. Int J Dent.

[30] Shetty SK, Kalia S, Patil K, Mahima VG (2005). Palatal rugae pattern in Mysorean and Tibetan populations. Indian J Dent Res.

[31] Kashima K (1990). [Comparative study of the palatal rugae and shape of the hard palatal in Japanese and Indian children]. Aichi Gakuin Daigaku Shigakkai Shi.

[32] Thomas CJ, Kotze TJ (1983). The palatal ruga pattern in six southern African human populations, part I: a description of the populations and a method for its investigation. J Dent Assoc S Afr.

[33] Buchtova M (2003). The development of palatal rugae in the European pine vole, Microtus subterraneus (Arvicolidae, Rodentia). Folia Zoo.

[34] Said KN, Abu Khalid AS, Farook FF (2020). Anatomic factors influencing dimensions of soft tissue graft from the hard palate. A clinical study. Clin Exp Dent Res.

[35] Breault LG, Fowler EB, Billman MA (1999). Retained free gingival graft rugae: a 9-year case report. J Periodontol.

[36] Karring T, Lang NP, Löe H (1975). The role of gingival connective tissue in determining epithelial differentiation. J Periodontal Res.

[37] Edel A, Faccini JM (1977). Histologic changes following the grafting of connective tissue into human gingiva. Oral Surg Oral Med Oral Pathol.

[38] Wolf HF, Rateitschak-Pluss EM (2005). Color atlas of dental medicine: periodontology. https://www.thieme.in/color-atlas-of-dental-medicine-periodontology.

[39] Cohen S (1994). Atlas of cosmetic and reconstructive periodontal surgery. https://books.google.com.sa/books/about/Atlas_of_Cosmetic_and_Reconstructive_Per.html?id=j8TNPAAACAAJ&redir_esc=y.

[40] Syed S, Alshahrani I, Alshahrani A, Togoo RA, Luqman M, Dawasaz AA (2016). Conversion of palatal rugae pattern to scanable Quick Response code in an Arabian population. J Dent Sci.

[41] Bing L, Wu XP, Feng Y (2014). Palatal rugae for the construction of forensic identification. Int J Morphol.

[42] Manjunath S, Bakkannavar SM, Kumar GP, Bhat VJ, Prabhu N, Kamath A, Babu YP (2012). Palatal rugae patterns among the Indians at Manipal, India. J Pharm Biomed Sci.

[43] Indira A, Gupta M, David MP (2012). Usefullness of palatal rugae patterns in establishing identity: preliminary results from Bengaluru city, India. J Forensic Dent Sci.

[44] Saadeh M, Ghafari JG, Haddad RV, Ayoub F (2017). Palatal rugae morphology in an adult mediterranean population. J Forensic Odontostomatol.

[45] Mittal S, Vyas P, Bhullar M, Singla D (2020). Arch length and palatal rugae: an adjunct in gender discrimination. Dent J Adv Stud.

